# Whole-Exome Sequencing Identifies Novel Genetic Variants Associated with Unexplained Neurodevelopmental Disorders in Children

**DOI:** 10.3390/ijms27020964

**Published:** 2026-01-18

**Authors:** Giancarlo Mancuso, Laura Serventi, Chiara Cocco, Francesco Lai, Consolata Soddu, Monica Marica, Caterina Mereu, Michela Lorrai, Gaia Maria Tosone, Federica Cannas, Giulia Nutile, Matteo Floris, Salvatore Savasta, Sabrina Giglio

**Affiliations:** 1 Medical Genetics Unit, Department of Medical Sciences and Public Health, University of Cagliari, 09124 Cagliari, Italy; 2 Pediatric and Rare Diseases Clinic, Microcitemico Hospital “A. Cao”, ASL 8 Cagliari, 09121 Cagliari, Italy; 3 Unit of Oncology and Molecular Pathology, Department of Biomedical Sciences, University of Cagliari, 09124 Cagliari, Italy; 4 Centre for Research University Services (CeSAR, Centro Servizi di Ateneo per la Ricerca), University of Cagliari, 09124 Cagliari, Italy; 5 Department of Biomedical Sciences, University of Sassari, 07100 Sassari, Italy; 6 Pediatric Clinic and Rare Diseases, Microcitemico Hospital, Department of Medical Science and Public Health, University of Cagliari, 09124 Cagliari, Italy; 7 Medical Genetics Unit, R. Binaghi Hospital, ASL 8 Cagliari, 09126 Cagliari, Italy

**Keywords:** neurodevelopmental disorders, whole exome sequencing, gene burden analysis

## Abstract

Neurodevelopmental disorders (NDDs) are a heterogeneous group of conditions characterised by impairments in cognition, motor function, behaviour, and social interaction. Their genetic basis is highly diverse, and next-generation sequencing has become central to improving diagnostic yield. We retrospectively analysed 94 paediatric patients (0–18 years) with NDDs referred to the Paediatric and Rare Diseases Clinic, Microcitemico Hospital “A. Cao,” between January 2019 and July 2024. Each patient underwent detailed clinical evaluation and whole-exome sequencing (WES). Variants were prioritised according to ACMG guidelines. Gene burden analysis of rare predicted loss-of-function variants was performed using the Cohort Allelic Sums Test to detect enrichment in NDD cases relative to controls. WES identified 12 pathogenic variants, 16 likely pathogenic variants, and 10 variants of uncertain significance. Autosomal dominant disorders were the most frequent (*n* = 35 patients), while autosomal recessive and X-linked dominant conditions were identified in a single case each. The findings of this study further highlight the importance of WES in identifying novel genetic variants and in providing explanations for previously unexplained NDD cases. Moreover, the Cohort Allelic Sums Test (CAST) demonstrated that rare variants are enriched in genes implicated in neuronal development in affected individuals.

## 1. Introduction

Neurodevelopmental disorders (NDDs) represent a heterogeneous group of conditions characterised by impairments in cognitive, motor, linguistic, and social development, typically manifesting during early childhood [1,2,3]. The clinical presentation of NDDs often spans multiple developmental domains and includes overlapping features, making accurate diagnosis challenging. Frequently, affected individuals present with comorbidities across different functional areas, further complicating both the diagnostic process and subsequent management [4]. Over the past decade, advances in diagnostic methods, enhanced clinical awareness, and the implementation of standardised developmental assessments have improved our ability to detect and characterise NDDs [5,6,7]. Nevertheless, distinction between different neurodevelopmental conditions remains complex due to shared phenotypic traits and the frequent co-occurrence of disorders [8,9]. From a biological standpoint, neurodevelopment is a dynamic and highly orchestrated process involving neuronal proliferation, migration, synaptic development, and myelination, extending from prenatal stages into adulthood [10,11,12,13]. Disruptions at any stage due to genetic, epigenetic, or environmental factors can result in enduring alterations in brain structure and function, manifesting clinically as neurodevelopmental disorders [14,15,16]. The aetiological landscape of NDDs is equally diverse. These conditions may present as syndromic or non-syndromic, depending on the presence or absence of additional systemic abnormalities [17,18,19]. Genetic causes of NDDs encompass a broad range of mechanisms, including chromosomal rearrangements, copy number variations (CNVs), single-nucleotide variants (SNVs), and epigenetic disruptions. To date, over 200 genes have been confirmed as causative for NDDs, and more than 1000 have been associated with these conditions [20]. Next-generation sequencing (NGS) technologies, particularly whole-exome sequencing (WES), have dramatically expanded our understanding of the genetic basis of NDDs and now serve as critical tools in their molecular diagnosis [21,22,23]. While whole-genome sequencing (WGS) offers the most comprehensive genomic coverage, including regulatory and non-coding regions, WES remains more accessible and cost-effective, with a more favourable interpretability profile in clinical settings [24,25]. Furthermore, trio-based WES remains a cornerstone in the evaluation of children with complex neurodevelopmental phenotypes, particularly when cytogenetic and targeted molecular analyses are inconclusive [26,27]. This study presents the clinical and molecular characterisation of a cohort of 94 patients with different NDDs, highlighting the substantial genetic heterogeneity and mechanistic complexity underlying these conditions. Through comprehensive genomic investigation, we report several novel variants that further expand the mutational spectrum associated with NDDs. Complementary gene burden analysis performed with the Cohort Allelic Sums Test (CAST) demonstrated that rare variants are enriched in particular genes in affected individuals, reinforcing their potential role in the underlying disease mechanisms.

## 2. Results

### 2.1. Whole-Exome Sequencing Results

**Figure 1:**
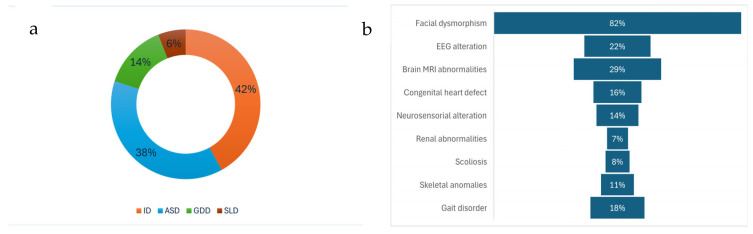
Graphical illustration showing (**a**) the distribution of the different neurodevelopmental disorders observed within the cohort and (**b**) the principal dysmorphic traits identified among affected individuals. ID: intellectual disability; ASD: autism spectrum disorder; GDD: global developmental delay; SLD: specific learning disorder; EEG: electroencephalogram.

A total of 94 paediatric patients underwent second-line genetic investigation using whole-exome sequencing, selected based on clinical presentation as outlined in the inclusion criteria. WES was performed using three testing strategies: 88 cases were analysed using a trio-based approach, 1 case was analysed as a duo (proband and one parent), and 5 cases underwent singleton analysis of the proband only. Among the 94 individuals who underwent WES, the primary neurodevelopmental diagnoses included intellectual disability in 40 patients, autism spectrum disorder in 36, global developmental delay in 12, and specific learning disorder in 6 patients (Figure 1). The cohort comprised children aged between 1 month and 14 years, with a median age at symptom onset of 24 months with an interquartile range of 1–4 years. The male-to-female ratio was 1.86:1, with 65% (61/94) of the patients being male. No cases of consanguinity were reported. A positive family history of neurodevelopmental disorders, defined as a first- or second-degree relative with a known history of NDDs, was documented in 27% (25/94) of cases. Premature birth was recorded in 11% of patients, including one born before 32 weeks of gestation and ten between 32 and 37 weeks (Table 1).

Comprehensive clinical evaluation revealed a high prevalence of multisystemic involvement (Table S5). As shown in Figure 1, facial dysmorphic features were noted in 82% of patients. Additional findings included EEG abnormalities in 22%, structural brain anomalies in 29%, congenital heart defects in 16%, neurosensory alterations in 14%, renal abnormalities in 7%, scoliosis in 8%, skeletal anomalies in 11%, and gait disturbances in 18% of the cohort.

**Figure 2:**
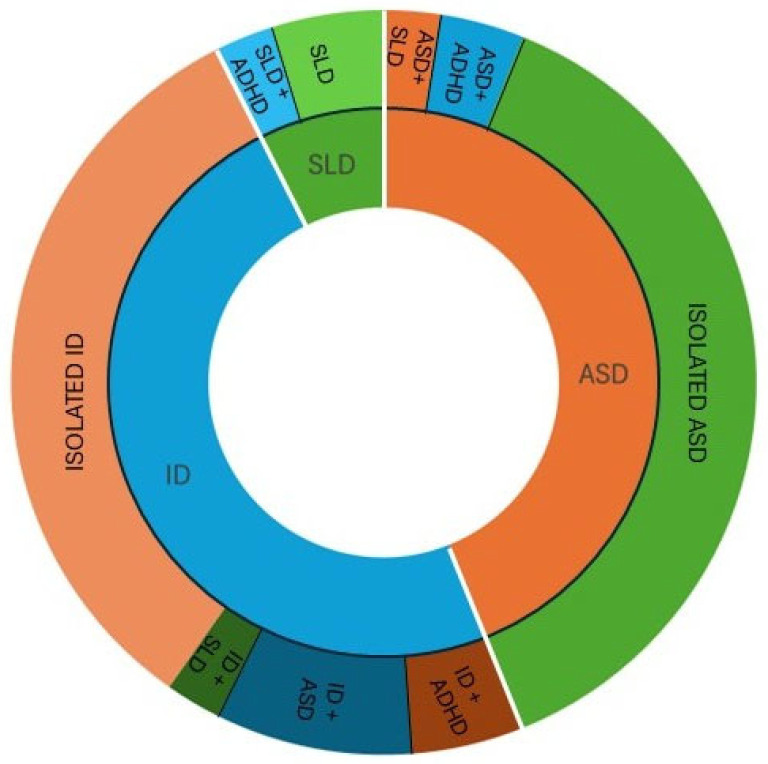
Graphical representation of comorbidities within the cohort. The internal circle reports the primary neurodevelopmental disorders diagnosed in the patients, while the external circle reports secondary neurodevelopmental conditions, illustrating the overlap of clinical phenotypes across disorders. ID: intellectual disability, ASD: autism spectrum disorder, SLD: specific learning disorder, ADHD: attention-deficit/hyperactivity disorder.

Analysis of comorbidities within the cohort revealed overlapping clinical phenotypes across neurodevelopmental conditions. Among patients with a primary diagnosis of autism spectrum disorder (ASD, *n* = 36), 5.6% also had a specific learning disorder and 8.3% had co-occurring attention-deficit/hyperactivity disorder. In patients with intellectual disability (ID, *n* = 39), 17.9% had coexisting ASD, 10.3% had ADHD, and 5.1% had SLD. Among patients with SLD (*n* = 6), 33% presented with comorbid ADHD (Figure 2).

Whole-exome sequencing identified 12 pathogenic variants, 16 likely pathogenic variants, and 10 different variants of uncertain significance (VUS). Most molecularly diagnosed cases followed an autosomal dominant inheritance pattern; only one case was consistent with autosomal recessive inheritance and one with an X-linked dominant disorder. Notably, the majority of variants have not been previously reported and are absent from population databases (Table 2; Supplementary Table S1). Within the autosomal dominant group, de novo variants predominated, with a single case of parental transmission.

### 2.2. CAST Analysis Results

To further investigate potential genetic contributors in undiagnosed cases, a Cohort Allelic Sums Test analysis was performed. This approach allows for the identification of genes enriched for rare predicted LoF variants in affected individuals compared to controls, thereby supporting gene discovery when individual variant analyses are inconclusive. The results of the CAST analysis revealed significant genetic associations with neurodevelopmental disorders by comparing the gene burden of LoF variants between cases and controls. Given the unique genetic characteristics of the Sardinian population [28], a subset of NDD patients was selected from the initial cohort of negative cases, excluding individuals with familial relatedness and/or non-Sardinian ancestry, to minimise potential confounding from shared genetic background or population stratification. These cases were compared with a reference dataset comprising 1246 unrelated healthy individuals of confirmed Sardinian ancestry, which was employed as a control cohort to ensure appropriate population matching. For each test, the CAST statistic was incorporated as a covariate in Firth’s logistic regression, with adjustments for sex, age, and family history. This analysis identified a total of 57 genes showing statistically significant associations (Supplementary Table S2). Following prioritization based on biological pathway relevance, clinical significance, and established involvement in neurodevelopmental processes, eight genes remained significantly associated (Supplementary Table S3). Among these, *BPTF* (OR = 4.59 × 10^14^; CI95% [5.08 × 10^14^–6.09 × 10^14^]; six cases, zero controls), *CEP295* (OR = 4.16 × 10^14^; CI95% [4.39 × 10^14^–5.57 × 10^14^]; five cases, zero controls), *HERC2* (OR = 5.48 × 10^14^; CI95% [5.99 × 10^13^–7.29 × 10^13^]; six cases, zero controls), and *PCLO* (OR = 4.16 × 10^14^; CI95% [4.39 × 10^14^–5.57 × 10^14^]; five cases, zero controls) represented the strongest associations. Across these selected genes, a total of 26 distinct patients were identified as harbouring at least one rare predicted loss-of-function variant. Across the prioritized genes, we identified at least one rare predicted loss-of-function variant in 26 distinct patients. Variants were recurrently detected in *BPTF* (six patients), *CEP295* (five patients), *COL27A1* (five patients), *COL6A2* (five patients), *HERC2* (seven patients), *NEB* (eight patients), and *PCLO* (five patients), with several individuals harbouring variants in more than one candidate gene.

## 3. Discussion

Over the past decade, advancements in genetic and genomic technologies have revolutionised clinical diagnostics, enabling the identification of numerous rare conditions that had previously remained undiagnosed for years. The widespread implementation of genetic testing, particularly the use of expanded gene panels and WES, has significantly improved our understanding of complex clinical presentations, including those underlying neurodevelopmental disorders [29]. In many cases, NDDs are characterised by substantial clinical and genetic heterogeneity, making the diagnostic process particularly challenging. WES has emerged as a powerful and cost-effective tool for uncovering pathogenic variants associated with rare monogenic diseases [30]. Its ability to comprehensively analyse the coding regions of the genome has provided an unprecedented opportunity to identify mutations involved in complex neurodevelopmental conditions, addressing the diagnostic gaps that often accompany these disorders [31].

In this study, we present data from a cohort of 94 paediatric patients with neurodevelopmental disorders who underwent comprehensive clinical and molecular characterisation using whole-exome sequencing, highlighting its value as a first-line diagnostic approach. We achieved an overall diagnostic yield of 39.4% (37/94), in line with previously reported data. Previous studies have documented variable success rates, with yields of approximately 25% in paediatric cohorts with isolated NDDs and up to 53% for cohorts in which NDDs are accompanied by additional conditions [32,33,34,35]. By sub-groups, this study showed a diagnostic yield of 7.4% in the ASD group, 22.3% in the ID group, and 9.6% in the GDD group. Statistical analysis revealed that the diagnostic yield for the ID/GDD group was significantly higher than that of the ASD group (*p*-value = 0.0028) and the SLD group (*p*-value = 0.0152). No significant difference was found between the ASD and SLD groups (*p*-value = 0.3164). Our cohort analysed approximately 94% of the cases using trio-based whole-exome sequencing. This approach significantly reduced the time required for genetic diagnosis and, in most instances, segregation analysis proved essential for definitively classifying variants as causative of disease. Variants were identified in 36 distinct genes, highlighting the extensive genetic heterogeneity of these conditions. Notably, as we expected, the majority of variants arose de novo. These variants were associated with autosomal-dominant and X-linked inheritance patterns in neurodevelopmental disorders, underscoring the critical role of the affected genes in central nervous system development and function. Most pathogenic variants identified were loss-of-function events that altered evolutionarily conserved amino acid residues known to be highly intolerant to variation [36]. Furthermore, the genes implicated in neurodevelopmental processes are strongly conserved across evolution, reflecting their essential role in multiple fundamental pathways [6]. In four cases, however, parental origin could not be determined due to the unavailability of one or both parental DNA samples.

The only inherited variant has been identified on the *ZMYM2* gene. This gene is a nuclear zinc finger protein that is localized to promyelocytic leukaemia nuclear bodies, where it forms part of transcriptional complexes acting as corepressors through interactions with various nuclear receptors and the LSD1–CoREST–HDAC1 chromatin-modifying complex [37,38]. The *ZMYM2* gene encodes a transcriptional regulator that plays a critical role in promoting and maintaining cell identity. Functional studies implicate *ZMYM2* in early embryonic development, particularly in the differentiation and lineage commitment of embryonic stem cells [37,39,40]. It is a ubiquitously expressed monomeric protein that functions predominantly as a transcriptional repressor, with activity detectable from the embryonic period onward. Moreover, *ZMYM2* has been shown to interact with key transcription factors involved in brain development, including forkhead box protein P1 (*FOXP1*) and homeobox protein SIX4 (*SIX4*), underscoring its potential role in neurodevelopmental regulatory networks [41,42].

To further assess the pathogenic potential of variants of uncertain significance identified in our cohort, we examined gene-level constraint metrics from gnomAD v4.0, focusing on the missense Z-score and probability of loss-of-function intolerance (pLI) of the implicated genes. All genes in our dataset showed a pLI = 1, highlighting a strong intolerance to variants causing protein truncation and supporting haploinsufficiency as a possible disease mechanism. All these genes also have a high missense Z-score (all genes have a value > 3, with a mean value of 5.49), indicative of a significant reduction in missense variation in the general population (Supplementary Table S4). The presence of a high pLI and high missense Z-score is indicative of the possibility that both loss of function and specific missense variants may be implicated in the pathogenesis of neurodevelopmental disorders [43].

Nevertheless, many cases remain without a molecular diagnosis. To better characterise these cases and try to reach a diagnosis, we analysed these samples using the CAST method. Our analysis using the CAST method revealed an enrichment of LoF variants in genes involved in several processes that are fundamental for proper neurological development. In particular, we focused on *BPTF*, *CEP295*, *COL27A1*, *COL6A2*, *HERC2*, *NEB*, and *PCLO*.

*BPTF*, encoding the largest subunit of the nucleosome remodelling factor complex, is a critical gene for chromatin accessibility and transcriptional programs that govern early embryogenesis and neural differentiation [44,45]. Loss-of-function or missense variants in *BPTF* have been associated with neurodevelopmental delay, intellectual disability, and dysmorphic features, underscoring the importance of epigenetic regulation in central nervous system development. These findings place *BPTF*-related disorders within the broader spectrum of chromatin remodelling syndromes, which are increasingly recognized as recurrent contributors to NDDs [46,47].

*CEP295* encodes for a centrosomal protein essential for centriole biogenesis, mitotic spindle organization, and ciliogenesis [48,49]. While pathogenic variants in *CEP295* are rarely reported in NDDs, defects in centrosomal proteins frequently disrupt neuronal proliferation and migration, often leading to microcephaly, structural brain abnormalities, and cognitive impairment. Its role in maintaining centrosome integrity therefore suggests a critical contribution to neurodevelopmental phenotypes when disrupted [50,51].

*COL27A1*, a fibrillar collagen crucial for skeletal development and cartilage formation, and *COL6A2*, a component of the collagen VI complex, are primarily associated with disorders showing muscular or skeletal involvement. However, both Steel syndrome (*COL27A1*) and Ullrich congenital muscular dystrophy (*COL6A2*) present with complex phenotypes that extend beyond musculoskeletal manifestations, also including neurodevelopmental involvement [52,53,54]. Extracellular matrix components emerged as significant modulators of neurodevelopment, highlighting the role of these extracellular matrix components in the functional organisation of neural circuits [55,56]. Cytoskeletal integrity is similarly critical for neurodevelopment. *NEB*, encoding nebulin, is primarily known for its role in sarcomere organization in skeletal muscle; however, cytoskeletal proteins are also essential for dendritic spine morphology, axonal transport, and neuronal structure. Disruption of *NEB* may therefore contribute to overlapping neuromuscular and neurodevelopmental phenotypes [57,58,59].

*HERC2*, a large E3 ubiquitin ligase, regulates DNA repair, cell cycle progression, and neurodevelopmental signalling pathways [60,61]. Biallelic mutations in *HERC2* give rise to a recognizable neurodevelopmental disorder characterized by global developmental delay, intellectual disability, autism spectrum features, hypotonia, and seizures. Dysfunction in protein homeostasis and DNA damage response likely contributes to impaired neuronal function and synaptic stability [62,63,64].

Finally, *PCLO*, encoding Piccolo, a core presynaptic scaffold protein, is integral to synaptic vesicle trafficking and neurotransmitter release [65,66,67]. Variants in *PCLO* have been linked to different neurodevelopmental conditions such as intellectual disability and epilepsy, illustrating how perturbations in synaptic organization and neurotransmission can manifest as neurodevelopmental phenotypes [68,69,70].

Collectively, these genes exemplify the diverse molecular pathways that can converge on similar clinical presentations in NDDs. Disruptions in chromatin remodelling, centrosome function, extracellular matrix composition, protein homeostasis, cytoskeletal dynamics, or synaptic signalling can each compromise neurodevelopment, resulting in overlapping cognitive, behavioural, and structural phenotypes.

Furthermore, although there was a notable enrichment of rare variants in these genes, each implicated in critical neurodevelopmental processes, none of the variants identified through the CAST analysis were classified as clearly causative. This observation suggests that while these genes may contribute to the genetic architecture of neurodevelopmental phenotypes, the variants detected in this cohort likely represent risk-modifying or subclinical alleles rather than fully penetrant pathogenic mutations. It also highlights the complex and multifactorial nature of neurodevelopmental disorders, where multiple rare variants with modest effects may collectively influence neurodevelopmental outcomes without a single variant being solely responsible for the phenotype.

One of this study’s primary limitations is the cohort’s heterogeneity, which is further influenced by the limited sample size and the monocentric nature of the study. The significant phenotypic diversity observed among patients may have influenced the selection criteria for genetic testing, contributing to potential selection bias in WES analyses. In the past, the need to establish stringent phenotypic criteria was driven by the necessity to optimise diagnostic yield, restricting testing to patients with highly suggestive clinical features indicative of a specific genetic condition. However, this approach carried the risk of excluding patients with atypical presentations or phenotypes overlapping multiple disorders, ultimately reducing the overall effectiveness of the diagnostic strategy. Considering recent developments and the previously discussed considerations, the selection criteria for WES should no longer be overly restrictive. Furthermore, as we have already highlighted, although WES is a powerful tool for detecting SNVs and indels, it remains less effective for identifying structural variations, balanced chromosomal rearrangements, deep intronic variants, and complex repetitive sequences [71,72]. The resolution of WES is insufficient for detecting small CNVs, particularly those spanning fewer than two exons, necessitating the complementary use of Chromosomal Microarray Analysis or WGS [73,74,75,76]. Another major challenge lies in variant interpretation. The presence of variants of uncertain significance underscores the complexity of determining their true clinical relevance. Although stringent filtering criteria and American College of Medical Genetics and Genomics (ACMG) guidelines were applied, some variants remain in a grey zone, requiring further functional validation to confirm pathogenicity. Over time, periodic reanalysis of WES data will be crucial as genetic databases expand and our understanding of disease mechanisms evolves [76]. Moreover, the lack of functional studies represents a weakness. While bioinformatics tools such as VarSome, ClinVar, Franklin by Genoox, and gnomAD provided predictive insights, true confirmation of variant pathogenicity demands experimental validation through transcriptomic or proteomic studies. Future research should integrate these approaches to strengthen genotype–phenotype associations.

## 4. Materials and Methods

### 4.1. Participants and Clinical Information

We conducted a retrospective study involving paediatric patients aged between 0 and 18 years who were referred to the Paediatric and Rare Diseases Clinic at the Microcitemico Hospital “A. Cao” (Cagliari, Italy) between January 2019 and July 2024. Inclusion criteria comprised the presence of a neurodevelopmental disorder, including intellectual disability, global developmental delay, autism spectrum disorder, attention-deficit/hyperactivity disorder, and/or specific learning disorders (SLDs); a comprehensive neuropsychiatric evaluation; and the absence of known non-genetic causes of NDDs. Only patients without a prior genetic diagnosis and with negative results from initial genetic investigations, such as array comparative genomic hybridisation (array-CGH) and Fragile X testing, were considered eligible. Individuals with a recognisable gestalt syndrome (e.g., Down syndrome, neurofibromatosis) were excluded. Informed consent was obtained from all parents or legal guardians for genetic testing and the disclosure of results. A total of 94 children from 90 unrelated families met the inclusion criteria and underwent whole-exome sequencing. For each patient, a detailed clinical assessment was performed, including a complete dysmorphological examination, a three-generation family history, and documentation of perinatal events and developmental milestones. WES analysis was conducted at the Molecular Genetics Unit of Binaghi Hospital in Cagliari.

### 4.2. Data Collection

The collected variables included the following: (1) sociodemographic: age, sex, and ethnicity; (2) family history: similar phenotype in parents, consanguinity, family history of neurodevelopmental disorder, and age of parents at the birth of their child; (3) patient’s clinical history: type of pregnancy (spontaneous or assisted reproduction), prenatal alterations, and neonatal pathology; (4) physical examination: weight, height and head circumference and dysmorphic features; (5) clinical features: degree of ID (mild, moderate, or severe), psychomotor development, congenital anomalies, associated neurodevelopmental disorders (language disorders, learning difficulties, attention-deficit/hyperactivity disorder (ADHD), and epilepsy); (6) cognitive: general intelligence assessed using the Wechsler intelligence scale for children, which evaluates a range of cognitive skills, including verbal comprehension, visual–spatial skills, fluid reasoning, working memory, and processing speed, and other aspects assessed include vocabulary, social cognition (emotion recognition, empathy), executive functions (working memory, inhibition, and cognitive flexibility), and attention; (7) diagnosis: result of exome sequencing, type of variant, inheritance pattern, genetic studies on parents, and age at diagnosis.

### 4.3. Sample Collection, WES Sequencing, Bioinformatic Analysis, and Variant Prioritization

Peripheral blood samples were formally collected and genetic analysis started after written informed consent was obtained from the parents of the proband. Peripheral blood samples were treated using the QIAamp DNA Blood Mini QIAcube Kit (Qiagen, Hilden, Germany) following the standardized kit. Whole-exome sequencing libraries were prepared using the KAPA HyperExome Plus Kit, KAPA Universal Adapter, and KAPA HyperCapture Bead Kit according to KAPA HyperCap Workflow v3.0 (Roche Sequencing, Boston, MA, USA). Paired-end exome sequencing with a read length of 150 bp was performed on the Illumina NextSeq550Dx Platform (Illumina Inc., San Diego, CA, USA) and achieved a medium target sequencing depth of 100X. Bioinformatic analysis was performed using the GenomeUp platform v.2(JuliaOmix™, Rome, Italy); sequence quality evaluation, low-quality reads, and adapter filtering were performed using FastP (version 0.20.3). Pre-processed reads were aligned against the GRCh38 reference genome using two alignment tools: bwa-mem2 (version 2.2.1) and DRAGEN (version 4.0.3) Germline. Variant calling was performed by using DRAGEN pipeline (Illumina Inc., San Diego, CA, USA) and following GATK best practice. To increase sensitivity, DeepVariant (version 1.5.0) was also used for variant calling using both GATK’s and DRAGEN’s BAM files. Subsequently, VCF files were merged and variants annotated using both Variant Effect Predictor (VEP, version 109) and SnpSift (version 5.1). A minimum depth coverage of 20X was considered suitable for analysis, based on the guidelines of the American College of Medical Genetics and Genomics (ACMG), and visualized by the Integrative Genome Viewer (IGV). The variants who passed the quality control criteria were summarized, cross-referenced, and clinically annotated using ClinVar classification. In silico prediction tools and databases were used for manual variant analysis, including the Human Gene Mutation Database (HGMD^®^).

### 4.4. Variant Inclusion and Classification

Strict variant filtering criteria were applied to ensure high accuracy and clinical relevance in the interpretation of sequencing data. Variants with a read depth below 20× were excluded from analysis, as were intronic and synonymous variants not known to affect splicing. Variant interpretation was guided by a combination of clinical and molecular parameters, including correlation with the patient’s phenotype to assess relevance to the suspected disorder, segregation analysis when parental DNA was available to determine inheritance patterns, and population frequency filtering. Only variants with a minor allele frequency (MAF) below 5% in the gnomAD database (http://gnomad.broadinstitute.org/) were retained for further consideration. All variants were classified according to the criteria established by the American College of Medical Genetics and Genomics (ACMG) [77]. To support variant interpretation and classification, several bioinformatics tools and publicly available databases were employed, including VarSome (https://varsome.com accessed on 20 November 2024), ClinVar (https://www.ncbi.nlm.nih.gov/clinvar accessed on 25 November 2024), Franklin by Genoox (https://franklin.genoox.com accessed on 27 November 2024), and gnomAD. Only variants classified as pathogenic (P), likely pathogenic (LP), or variants of uncertain significance (VUS) were reported.

### 4.5. Disease-Causing Variants for Gene Burden Testing

The gene-based burden for rare variants in cases versus controls was quantified using the Cohort Allelic Sums Test (CAST) statistic, considering the presence of at least 5 rare, predicted loss-of-function (LoF) variants, irrespective of the mode of inheritance. This analysis incorporated an additional variant quality control step in which variants observed in at least one case were excluded if present in at least one control in any zygosity state (heterozygote, homozygote, or compound-heterozygote) to mimic a Mendelian fully penetrant-like disease model. The gene burden association was then assessed using either a binary case–control status with a right-tailed Fisher exact test on the CAST statistic and a Firth logistic regression model, using the CAST statistic as a covariate, to correct for unbalanced case–control datasets with rare events. In the analysis of the rare disease component, Firth logistic regression was adjusted for age, sex, and family history. Patients who were related were removed from the analysis to avoid confounding due to familial relatedness. Finally, multiple testing correction was applied using the Benjamini and Hochberg method, with a false discovery rate-adjusted P-value threshold of 0.5% for statistically significant disease–gene associations to be prioritized for further investigation. Gene Ontology terms were used for pathway analysis of results from rare variant burden tests.

### 4.6. Statistical Analysis

A two-tailed Fisher’s exact test was used to compare the diagnostic yield between the different NDD categories. *p*-values were calculated for each pairwise combination of NDDs represented in the cohort to identify significant differences in diagnostic probability.

## 5. Conclusions

This study highlights the value of trio-based WES in the genetic diagnosis of NDDs, demonstrating its efficiency in identifying causative variants and informing personalized patient management. However, despite these promising results, the scope of the study is restricted by the cohort size and limitations in the clinical setting of the patients studied. The aim is to expand this work by analysing larger and more diverse patient cohorts. Incorporating additional cases will improve statistical power, enhance the identification of rare and novel variants, and refine genotype–phenotype correlations. Furthermore, integrating WES with complementary approaches such as whole-genome long-read sequencing, epigenomic profiling, and transcriptomic analyses will help fill current diagnostic gaps. Finally, efforts should be directed to improve bioinformatics tools for variant interpretation, expand functional validation studies, and improve accessibility to genomic technologies. A multidisciplinary strategy that combines genomic data with in-depth clinical phenotyping will be essential to improve the diagnosis and management of NDDs.

## Figures and Tables

**Table 1:** Demographic and neurodevelopmental characteristics of the cohort. Age at symptom onset is reported as median.

Clinical Findings	Value	%
Male	61	65%
Female	33	35%
Age at symptom onset (months)	24	
Positive family history	25	27%
Birth at term	83	88%
Premature birth	11	12%
Neurodevelopmental disorder		
Autism spectrum disorder	36	38%
Intellectual disability	39	42%
Global developmental delay	13	14%
Specific learning disorder	6	6%

**Table 2:** Genomic variants identified through WES in our group of patients. ID: intellectual disability; ASD: autism spectrum disorder; GDD: global developmental delay; het: heterozygous; AD: autosomal-dominant; AR: autosomal-recessive; XLD: X-linked-dominant; N/A: parent samples not available; pat: paternal; mat: maternal.

Case n.	Phenotype	Gene (Transcript)	Variant	Zygosity	Inheritance	Segregation
2	GDD	*NIPBL* (NM_133433.4)	c.6056T>C p.(Leu2019Pro)	het	AD	NA
5	ID	*DLG4* (NM_001321075.3)	c.14_15delTG p.(Cys5Tyrfs*12)	het	AD	de novo
6	GDD	*MED13L* (NM_015335.5)	c.700A>G, p.(Lys234Glu)	het	AD	de novo
16	ID	*KCNA2* (NM_004974.4)	c.889C>T p.Arg297Trp	het	AD	de novo
17	ID	*PPP2R1A* (NM_014225.6)	c.544C>T p.(Arg182Trp	het	AD	de novo
18	ID	*SETD5* (NM_001080517.3)	c.2750delT p.(Leu917fs)	het	AD	de novo
23	GDD	*PPP2R5D* (NM_006245.4)	c.592G>A p.(Glu198Lys)	het	AD	de novo
25	ASD	*ZMYM2* (NM_197968.4)	c.3729_3730del p.(Phe1244*)	het	AD	pat
26	ID	*ATP1A1* (NM_000701.8)	c.1181A>T p.(Asp394Val)	het	AD	de novo
27	ID	*HNRNPK* (NM_031263.4)	c.1241G>A p.(Arg414His)	het	AD	de novo
30	ASD	*MECP2* (NM_001110792.2)	c.952C>T p.(Arg318Cys)	het	AD	de novo
31	ID	*MED13L* (NM_015335.5)	c.1768C>T p.(Gln590*)	het	AD	de novo
41	ID	*CHD2* (NM_001271.4)	c.762_764del p.(Asp254del)	het	AD	de novo
42	ID	*SCN8A* (NM_001330260.2)	c.2811G>A p.(Trp937*)	het	AD	de novo
43	ID	*SCN8A* (NM_001330260.2)	c.2811G>A p.(Trp937*)	het	AD	de novo
44	ASD	*GRIN2A* (NM_001134407.3)	c.395c>T p.(Ser132Phe)	het	AD	de novo
45	ASD	*TCF12* (NM_207037.2)	c.686-1G>C	het	AD	de novo
50	ID	*POU4F1* (NM_006237.4)	c.486_487insGG p.(Pro163Gly)	het	AD	de novo
54	ID	*ANKRD11* (NM_013275.6)	c.1903_1907del (p.Lys635fs)	het	AD	de novo
55	GDD	*PPP2R5D* (NM_006245.4)	c.592G>A p.(Glu198Lys)	het	AD	de novo
59	ID	*SMARCA2* (NM_003070.5)	c.1574G>A p.(Arg525His)	het	AD	de novo
60	ASD	*ASH1L* (NM_018489.3)	c.4579C>T p.(Arg1527*)	het	AD	de novo
63	ID	*AUTS2* (NM_015570.4)	c.1603_1626del p.(His535-Thr542del)	het	AD	de novo
64	GDD	*ITPR1* (NM_001378452.1)	c.1554+6T>G	het	AD	N/A
67	ASD	*TRAPPC9* (NM_001374682.1)	c.307A>T p.(Lys103*)	het	AR	mat
			c. 610 C>T p.(Arg204Cys)	het	AR	pat
71	ID	*SETBP1* (NM_015559.3)	c.1075C>T p.(Gln359Ter)	het	AD	de novo
74	GDD	*SMC1A* (NM_006306.4)	c.170G>A p.(Arg57Gln)	het	XLD	de novo
77	GDD	*KMT2A* (NM_001197104.2)	c.3853C>T p.(Gln1285*)	het	AD	de novo
79	ID	*PRRT2* (NM_145239.3)	c.649del p.(Arg217GlufsTer12)	het	AD	de novo
80	ASD	*SETD1A* (NM_014712.3)	c.644_645delinsCC p.(Glu215Ala)	het	AD	de novo
83	ID	*CHD5* (NM_015557.3)	c.2842G>T p.(Val948Phe)	het	AD	N/A
84	GDD	*CHD5* (NM_015557.3)	c.2842G>T p.(Val948Phe)	het	AD	N/A
85	ID	*GRIA2* (NM_001083619.3)	c.857C>G p.(Pro286Arg)	het	AD	de novo
88	ID	*SMARCD1* (NM_003076.5)	c.77C>T p.(Ala26Val)	het	AD	de novo
89	ID	*ANKRD11* (NM_013275.6)	c.5806G>T p.(Glu1936*)	het	AD	de novo
91	GDD	*KAT6A* (NM_006766.5)	c.3553C>T p.(Gln1185Ter)	het	AD	de novo
94	ID	*PTEN* (NM_000314.8)	c.1003C>T p.(Arg335*)	het	AD	de novo

## Data Availability

The original contributions presented in this study are included in the article/Supplementary Material. Further inquiries can be directed to the corresponding authors.

## Supplementary Materials

The following supporting information can be downloaded at: https://www.mdpi.com/article/10.3390/ijms27020964/s1.

## Abbreviations

The following abbreviations are used in this manuscript:

ACMG	American College of Medical Genetics and Genomics
ADHD	Attention-Deficit/Hyperactivity Disorder
Array-CGH	Array Comparative Genomic Hybridisation
ASD	Autism Spectrum Disorder
CAST	Cohort Allelic Sums Test
CNVs	Copy Number Variations
GDD	Global Developmental Delay
ID	Intellectual Disability
LoF	Loss-Of-Function
NDDs	Neurodevelopmental Disorders
NGS	Next-Generation Sequencing
pLI	Loss-Of-Function Intolerance
SLD	Specific Learning Disorders
SNVs	Single-Nucleotide Variants
WES	Whole-Exome Sequencing
WGS	Whole-Genome Sequencing
